# Delay in *Aceria guerreronis* infestation may reduce necrosis and fruit drop in coconut trees

**DOI:** 10.1007/s10493-026-01114-1

**Published:** 2026-02-11

**Authors:** Girleide Vieira de França-Beltrão, Vanessa Farias da Silva, Antônio de Almeida Paz Neto, José Wagner da Silva Melo, Manoel G. C. Gondim Junior

**Affiliations:** 1 Departamento de Agronomia - Entomologia, Universidade Federal Rural de Pernambuco, Rua Dom Manoel de Medeiros, Recife, 52171-900 PE Brasil; 2 Recife, PE Brasil; 3 Departamento de Fitotecnia - Agronomia, Universidade Federal do Ceará, Av. Mister Hull, Fortaleza, 60356-001 CE Brasil; 4 Departamento de Zoologia – Centro de Biociência, Universidade Federal de Pernambuco, Avenida Professor Moraes Rego, Recife, 50670-420 PE Brasil

**Keywords:** Coconut palm, Coconut mite, Fruit age, Damage, Fruit abortion

## Abstract

The coconut mite, *Aceria guerreronis*, colonizes the fruit perianth, a region covered by floral bracts, which hinders its control. Its attack causes epidermal necrosis, fruit deformation, and premature fruit drop, with yield losses ranging from 10% to 70%. Infestations generally begin in fruit up to three months old. Since the age of the fruit at the time of infestation can influence not only the severity of damage but also fruit abortion, these aspects were investigated through field experiments. Fruit aged 2 to 5 months were artificially infested with *A. guerreronis*, and every 15 days, necrotic area and number of aborted fruit were quantified until harvest (≈ 8 months). Both damage severity and abortion rate were significantly higher in younger fruit (2–3 months) than in older fruit (4–5 months); however, damage severity had no direct influence on abortion. These results indicate that the extent of necrotic lesions is not a determining factor for fruit abortion. Despite significant damage observed in some infested fruit, many remained attached to the plant and continued to develop. Others, however, aborted without any necrotic lesions. These findings suggest that other factors, such as physiological mechanisms of tolerance or compensation by the host plant, may influence fruit retention. Understanding these dynamics is essential to improve pest management strategies and avoid overestimating potential yield losses associated with mite infestation.

## Introduction

Pest incidence in *Cocos nucifera* L. is one of the main limiting factors for its production (Howard et al. 2001; Fontes and Ferreira 2006). The coconut mite, *Aceria guerreronis* Keifer (Acari: Eriophyidae), is considered one of the most significant pests of coconut crops in the Americas, Africa, and parts of Asia (Moore and Howard 1996; Fernando et al. 2002; Navia et al. 2013). Colonies of *A. guerreronis* develop beneath the perianth of coconut fruit (Howard and Rodriguez 1991; Aratchige et al. 2007), causing necrosis and epidermal deformation (Moore et al. 1989; Haq et al. 2002; Paul and Mathew 2002; Negloh et al. 2011). Infested fruit that reach harvest maturity show varying degrees of necrotic damage depending on plant variety, climatic conditions, control practices, and other factors (Mariau and Julia 1970; Suarez 1991; Seguni 2002; Navia et al. 2005; Ramaraju et al. 2005; Fernando and Aratchige 2010; Negloh et al. 2011; Rezende et al. 2016). Fruit necrosis increases with the growth of the mite’s population, with peak densities (averaging 943 mites per fruit) observed in fruit (dwarf green variety) aged between 3 and 4 months (Galvão et al. 2011). This population peak typically occurs when approximately 16% of the fruit surface is necrotic. However, even as the mite population declines, necrosis continues to progress, often affecting more than 50% of the epicarp, rendering the fruit unsuitable for the fresh market (Rezende et al. 2016).

Infestation by *A. guerreronis* is infrequent in younger fruit (1–2 months old) (Negloh et al. 2010, 2011), likely due to the limited space available for mites to access the perianth of these fruit, as shown by Lima et al. (2012). Adult females of *A. guerreronis* (36–52 μm thick) can readily reach the perianth of fruit aged 3 months or older (Aratchige et al. 2007; Lima et al. 2012). Nevertheless, fruit aged 5 months or older rarely exhibit recent damage by *A. guerreronis* (chlorotic white-yellowish spots), suggesting that fruit at this developmental stage may not be suitable for colony establishment (Galvão et al. 2011), or may even be avoided by the mites.

Premature fruit drop in coconut palms has also been attributed as a direct consequence of mite infestation (Moore et al. 1989; Seguni 2002; Nair 2002; Rethinam et al. 2003). However, more recent studies suggest that fruit drop may also be an indirect consequence, facilitated by necrosis caused by the mite, which provides entry points for secondary infestation by other organisms (Lakshmanan and Jagadeesan 2004; Paz-Neto et al. 2020, 2022). While the causal relationship behind premature fruit abortion remains debated, *A. guerreronis* infestation is known to directly and/or indirectly increase fruit abortion. Nonetheless, no studies to date have established a direct correlation between the extent of epidermal necrosis caused by *A. guerreronis* and fruit abortion.

Losses caused by *A. guerreronis* have been estimated in several countries using diverse methodologies, making direct comparisons between studies difficult. Available data show that this pest can reduce productivity by 10% to 70%, depending on the producing region (Navia et al. 2013; Rezende et al. 2016). These estimates typically consider reductions in fruit size and weight, which in turn lower the yield of liquid endosperm (coconut water), solid endosperm (copra), and coir fiber (Paul and Mathew 2002; Seguni 2002; Ramaraju et al. 2005; Negloh et al. 2011). Mite-infested palms consistently produce lower yields (Doreste 1968; Moore et al. 1989; Seguni 2002; Wickramananda et al. 2007; Rezende et al. 2016). Although these studies highlight the pest’s negative impact on fruit production, none has directly correlated damage severity (percentage of necrotic area) or *A. guerreronis* population levels with yield reduction.

The impact of *A. guerreronis* on fruit of different ages still requires targeted investigation, as control practices such as spraying all plant bunches may result in unnecessary costs, increased acaricide use per area, negative effects on native fauna, management failures, and other issues. Therefore, we conducted a field experiment in which fruit aged between 2 and 5 months were artificially infested. We quantified the severity of damage (chlorosis and/or necrosis) and monitored fruit development and/or abortion until harvest (approximately 8 months of age for the dwarf green variety). We also analyzed the relationship between *A. guerreronis*-induced damage severity and premature fruit abortion across different fruit age groups.

## Materials and methods

### Estimation of population density for artificial infestation

**Fig. 1:**
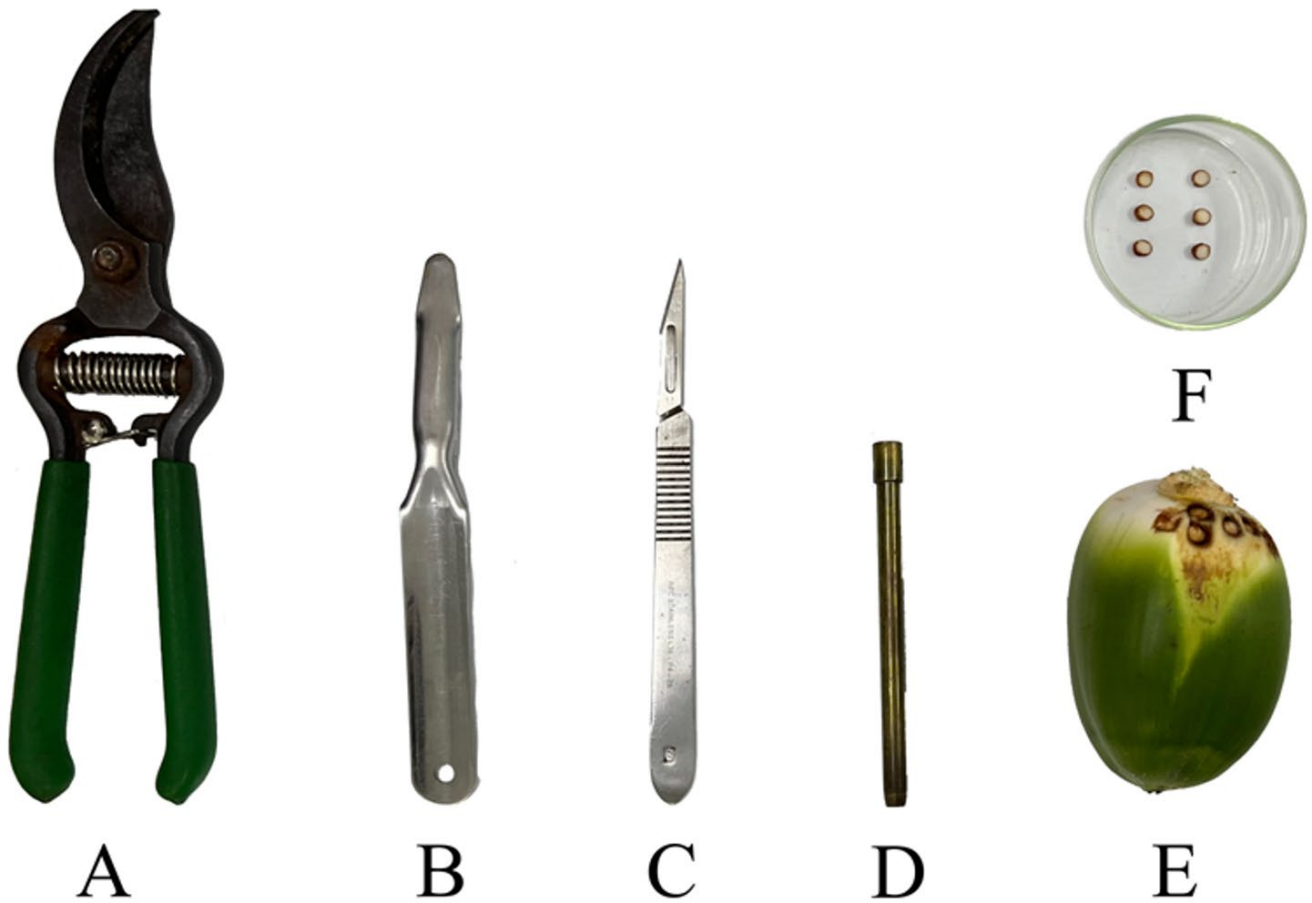
Methodology for extracting a fragment of the meristematic zone from *Aceria guerreronis*-infested fruits for artificial infestation of *Cocos nucifera* fruits in the field. Floral bracts were carefully removed using pruning scissors and a spatula. Mite colonies were then located, and a 4 mm diameter fragment of the perianth epicarp was extracted from the center of each colony using a punch. We inserted half of this perianth fragment under the inner bracts of the coconut fruit. **A**: pruning scissors; **B**: spatula; **C**: scalpel; **D**: puncher; **E**: mite colony; **F**: epicarp disk containing mites

Ten-year-old native hybrid coconut palms (*C. nucifera*) exhibiting fruit necrosis exclusively caused by *A. guerreronis* were selected in the municipality of Igarassu, Pernambuco, Brazil (7°50′S, 34°54′W). Fruit from bunches aged 3 to 4 months (from leaves 13 and 14, according to the phyllotaxy of coconut palms; Sobral 1998) were collected and transported to the laboratory, where they were stored in a refrigerator at approximately 10 °C. Floral bracts were carefully removed using pruning scissors and a spatula. Mite colonies were then located, and a 4 mm diameter fragment of the perianth epicarp was extracted from the center of each colony using a punch. Each fragment was examined under a stereomicroscope, and all active stages of *A. guerreronis* were counted. This process was repeated 20 times, yielding an average of 166 ± 22 mites per fragment (Fig. 1A–F).

**Damage severity and fruit abortion caused by** ***A. guerreronis*** **infestation**.

**Fig. 2:**
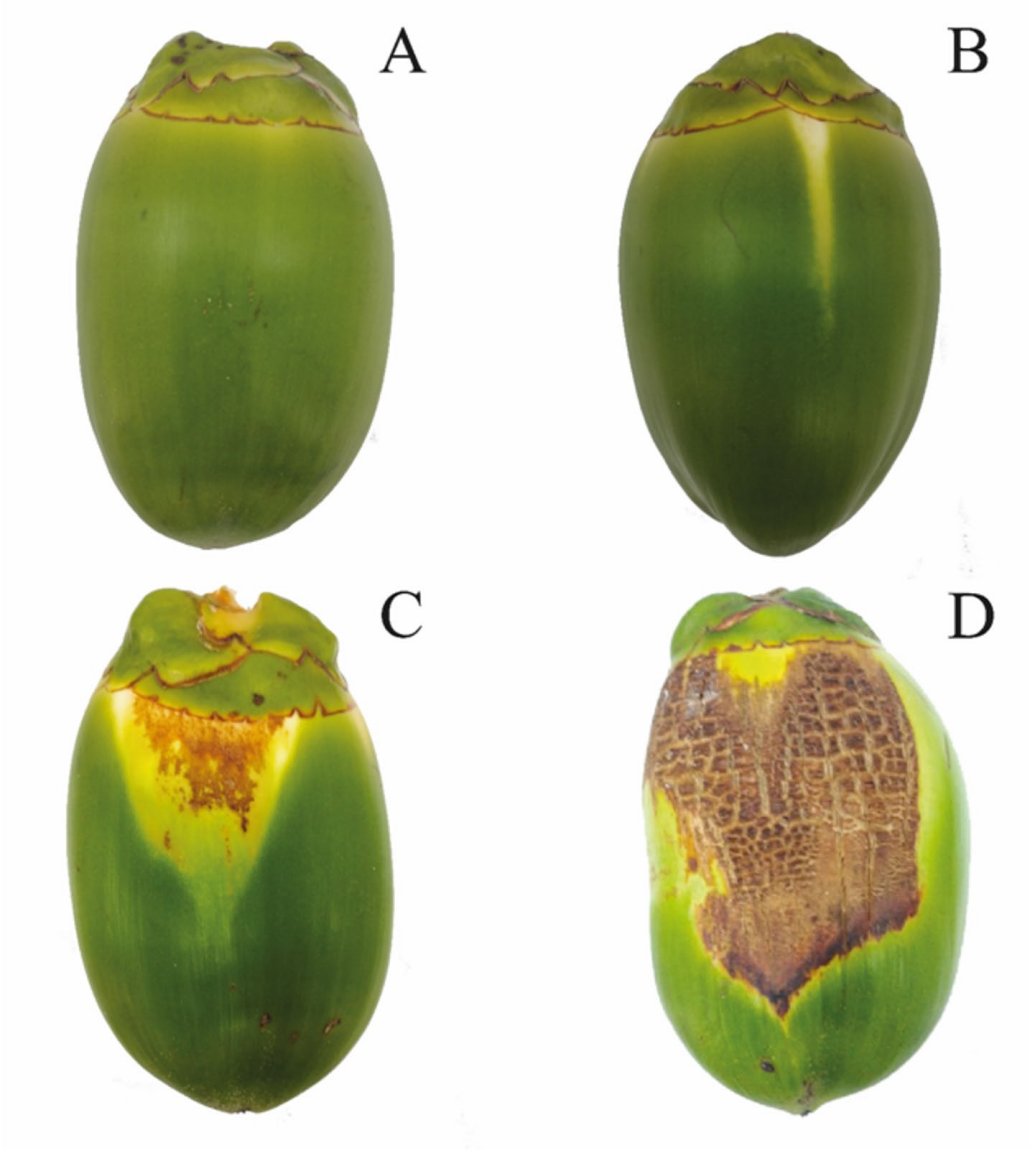
*Cocos nucifera* fruits and progression of damage intensity caused by *Aceria guerreronis*. **A**: fruit without damage; **B**: fruit with initial damage (chlorosis); **C**: fruit with intermediate damage (necrosis); **D**: fruit with advanced damage (necrosis and deformation)

**Fig. 3:**
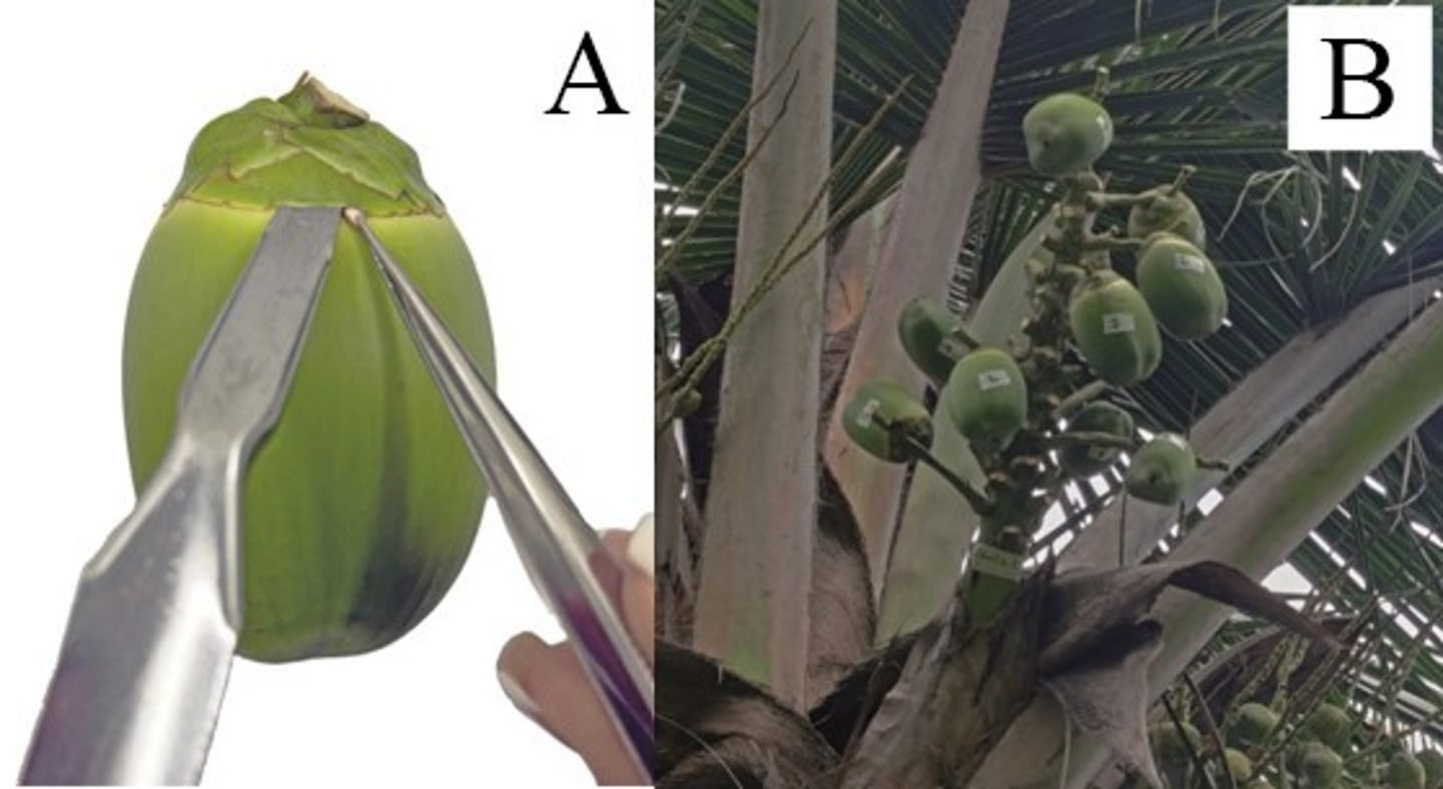
*Cocos nucifera* fruits artificially infested in the field with *Aceria guerreronis*. **A**: insertion of the infested epidermal fragment into the perianth of undamaged fruits; **B**: selected, marked, and infested fruits in the field

Ten-year-old dwarf green coconut palms whose fruit showed no visible symptoms of *A. guerreronis* damage (chlorosis and/or necrosis; Fig. 2A) were selected at the Department of Agronomy, Universidade Federal Rural de Pernambuco (8º01′S, 34º94′W). The experiments were repeated at different times between September 2012 and May 2015, with average temperatures ranging from 22.6 to 30 °C and relative humidity from 42.9 to 72.2%. For artificial infestation, four palms were selected. From each palm, 10 fruit were chosen from bunches aged between 2 and 5 months, totaling 40 fruit per plant and 160 fruit overall. All selected fruit were tagged, recording both the bunch age and palm number. Only the selected fruit were kept on the bunches; all others were removed by cutting their spikelets at the proximal end to minimize effects on resource allocation and, consequently, reduce bias in fruit abortion (Fig. 3A).

Using a spatula, the bracts of each fruit were gently lifted. A fragment of the perianth infested by *A. guerreronis* was collected as described in the previous section. Then, we inserted half of this fragment under the inner bracts (i.e., the bracts in direct contact with the epicarp; see Lawson-Balagbo et al. 2007), approximately 83 active mites. (Fig. 3B).

For the non-infested control treatment, an additional four mite-free palms were selected. From each of these, only 10 fruit from bunches aged 2 months were selected (totaling 40 fruit). Fruit tagging and identification followed the same procedure as described above.

**Fig. 4:**
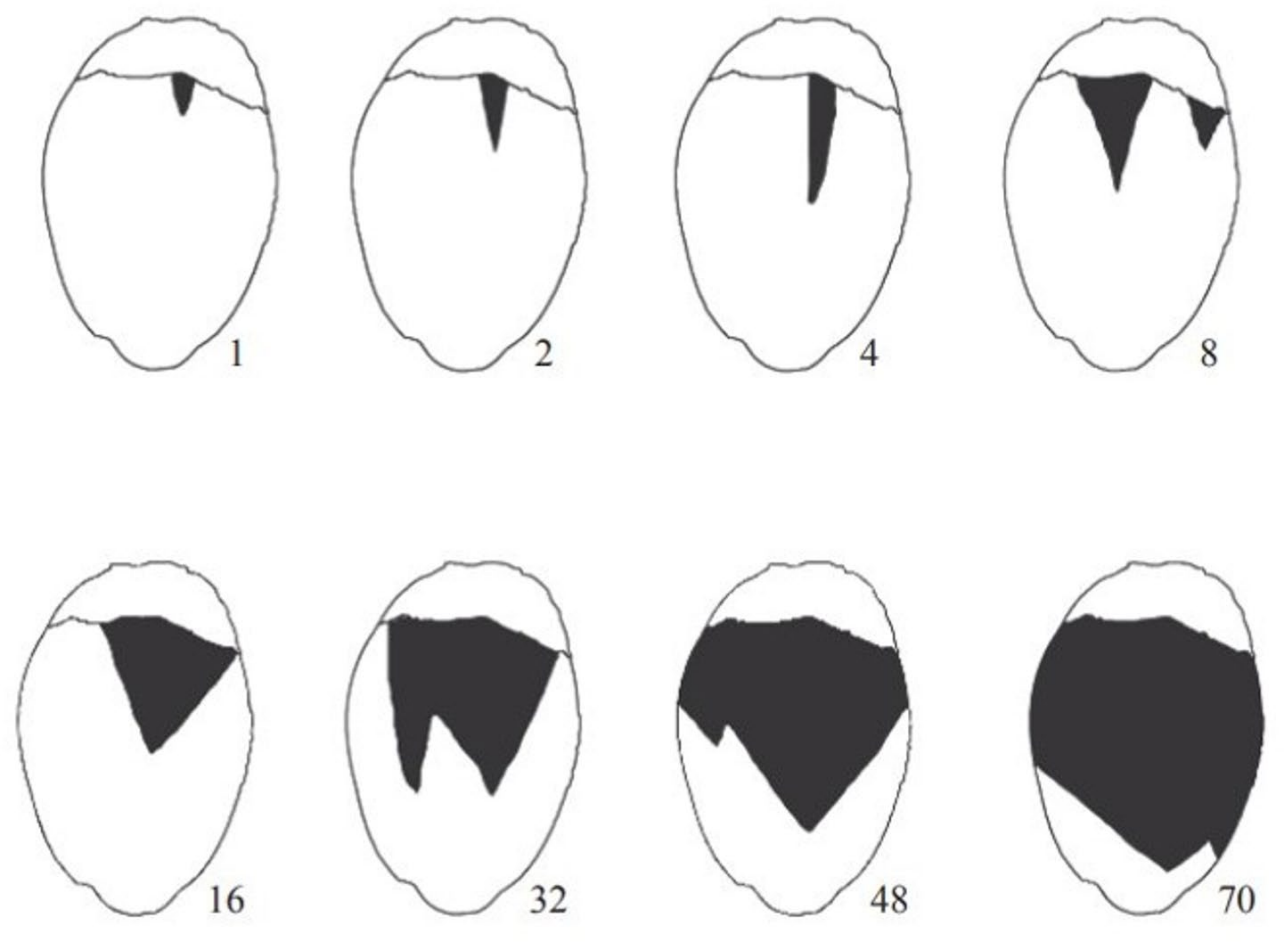
Diagrammatic scales proposed by Galvão et al. (2008) for estimating the population density of *Aceria guerreronis*. The values indicated next to the figures represent the percentage of necrotic area

All fruit aborted or not were assessed biweekly (at 15, 30, 45, 60, 75, 90, 105, and 120 days). Each fruit was visually evaluated for damage severity (chlorosis and/or necrosis) using the diagrammatic scale developed by Galvão et al. (2008) (Fig. 4). According to this scale, lesions are assessed on each face of the fruit and may range from 1% to 70%. Monitoring continued until the fruit either aborted or reached harvest maturity.

We compared fruit abortion progression and *A. guerreronis* damage severity between artificially infested and non-infested fruit, considering only the data from 2-month-old bunches. Additionally, we evaluated the effect of fruit age at the time of infestation on abortion and damage severity. In this analysis, each bunch age was considered a treatment, each bunch a replicate, and each fruit a pseudo replicate. We conducted the experiment on 6 bunches for each age.

## Statistical analyses

The data were subjected to regression analysis using TableCurve 2D software (Systat, San Jose, CA, USA), with evaluation times (15, 30, 45, 60, 75, 90, 105, and 120 days) as the independent variable, and the mean levels of damage severity (chlorosis and/or necrosis) or the percentage of fruit abortion (for 2-month-old fruit) as dependent variables. This analysis was performed for both artificially infested and non-infested coconut fruit. Statistically significant regression models (*P* < 0.05) were selected based on the principle of parsimony, favoring models with high F and R² values. For each model, residual distributions were examined to verify the assumptions of parametric analysis.

The damage level represents the ratio between the area of ​​the fruit with or without mite injury; therefore, these are proportion data that vary between 0 and 1. The mean levels of damage severity (chlorosis and/or necrosis) in non-aborted fruit from bunches artificially infested at 2 to 5 months age were analyzed using generalized linear mixed models (GLMMs) with a beta distribution and repetition (different bunchs of the same age) as a random variable (glmmTMB(Damage ~ Age + (1|Repetition), family= ordbeta), followed by post-hoc mean separation using Tukey’s test for comparison among ages. The number of aborted fruit from artificially infested bunches at 2 to 5 months of age was compared using generalized linear mixed models (GLMMs) with a binomial distribution and repetition (different bunchs of the same age) as a random effect variable (glmmTMB(Abortion ~ Age + (1|Repetition), family= binomial(link = “logit”), followed by post-hoc mean separation using Tukey’s test for comparison among ages. Aborted fruit were also assessed for damage severity (chlorosis and/or necrosis), being categorized according to the diagrammatic scale developed by Galvão et al. (2008). After categorization, data from each bunch were subjected to a chi-square test (compare the observed and expected frequency of event occurrence) to evaluate the possible relationship between the severity of *A. guerreronis* damage and fruit abortion.

## Results

**Fig. 5:**
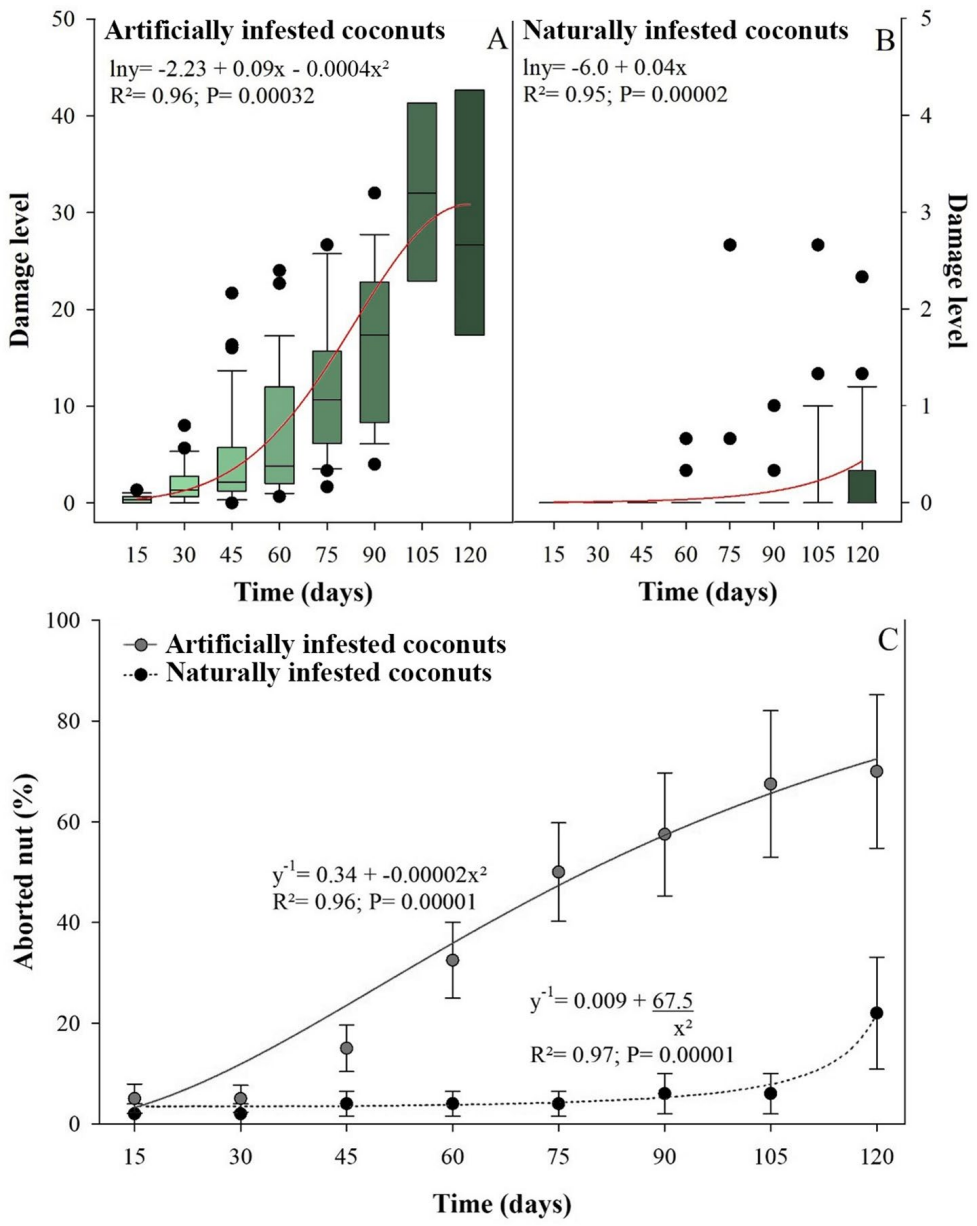
Damage intensity (chlorosis and/or necrosis) or fruit abortion caused by *Aceria guerreronis* over time. The boxplots represent the level of damage caused by *A. guerreronis* in coconut fruit at 2 months of age over time. The variation in the data is represented by the whiskers and the points (outliers) of each boxplot. **A**: artificially infested coconuts; **B**: naturally infested coconuts. **C**: curves of coconut fruits abortion, whether artificially or naturally infested, over time. Alpha equal to 5% significance

At the first assessment, 15 days after the artificial infestation of 2-month-old fruit, initial symptoms of *A. guerreronis* damage were already visible, characterized by triangular chlorotic spots (Fig. 2B). A slow increase in damage severity (% of necrotic fruit area) was observed during the first two evaluations (15 to 30 days) on artificially infested fruit. In subsequent evaluations (45–90 days), damage severity increased significantly, showing rapid progression with a transition from chlorosis to necrosis and a considerable expansion of the affected area, often accompanied by fruit deformation (Figs. 2C–D). From the seventh evaluation (90 days) onward, there was a slow increase in the necrotic area, stabilizing the growth in damage severity, where the average severity was 30% in the final assessment (120 days after the start of the experiment). Overall, the increase in necrotic area over time in artificially infested fruit was adjusted to a quadratic curve (degree 2) (R² = 0.96; *P* < 0.001) (Fig. 5A).

In the naturally infestation group, the symptoms of *A. guerreronis* damage began to appear in the third evaluation (45 days after the start of the experiment). This delay in infestation resulted in a slow increase of mite damage until the harvest period (120 days after the start of the experiment). Thus, the increase in necrotic area over time in naturally infested fruit was adjusted to a linear curve (R² = 0.95; *P* < 0.001). Furthermore, the average severity of damage (necrosis) in naturally infested fruits never exceeded 5% of the fruit’s surface area (Fig. 5B).

Artificial infestation of young fruit (2 months old) increased premature fruit drop over time, with a reduction of approximately 70% in the number of fruits that reached harvest time. In artificially infested fruits, we observed that the fruit abortion data showed a constant response over time, fitting a linear curve (R² = 0.96; *P* < 0.001) (Fig. 5C), unlike the damage level data.

In naturally infested fruits, there was less premature fruit drop, an average reduction of 20% up to harvest time. Natural infestation by *A. guerreronis* caused low abortion rates in young fruits, with a rapid increase in older ones. This response over time fitted the linear curve (R² = 0.97; *P* < 0.001) (Fig. 5C), in the same way as the damage level data.

**Fig. 6:**
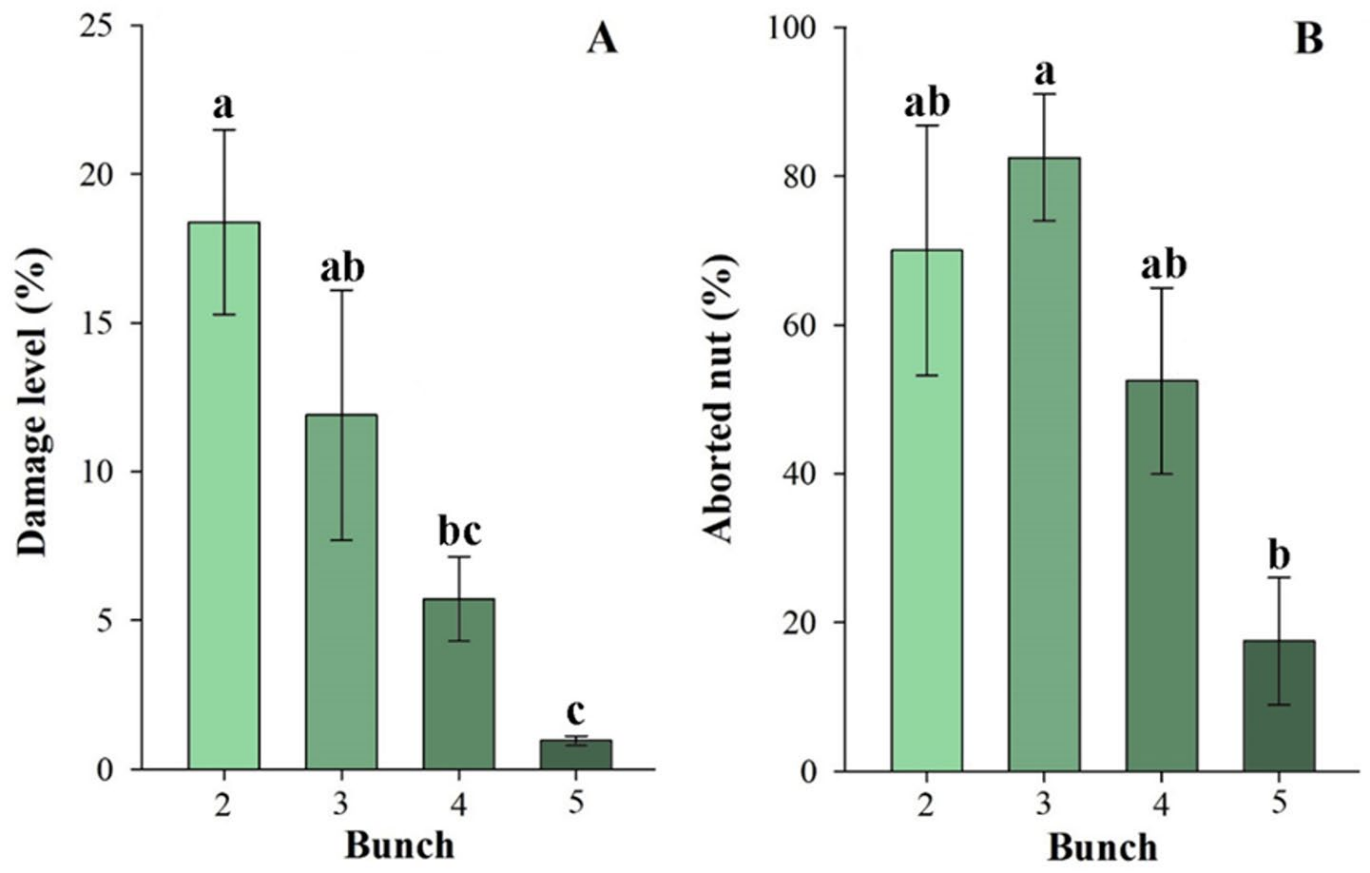
Damage intensity (chlorosis and/or necrosis) or fruit abortion caused by *Aceria guerreronis* in fruits artificially infested at 2 to 5 months of age, at harvest time. **A**: damage intensity; **B**: abortion percentage

The generalized mixed model explained 42% of the variability in damage level between different ages (fixed effects - Marginal R² = 0.420), and 55% considering also the variability between bunches (random effect - Conditional R² = 0.548). The comparison of means showed that the older fruits (4 and 5 months old) presented a lower level of mite damage at harvest time (120 days after the start of the experiment) compared to the younger ones (2 and 3 months old). However, statistically, no difference was observed between the damage level for fruit aged 3 and 4 (age3 - age4: *P* = 0.8393), and a marginally significant difference between ages 3 and 5 (age3 – age5: *P* = 0.0401) (Fig. 6A).

The generalized mixed model explained 6.7% of the variability in abortion between different ages (fixed effects - Marginal R² = 0.067), and 14.8% considering also the variability between bunches (random effect - Conditional R² = 0.148). Similarly, abortion rates were lower in older fruit (5 months old), but, the abortion rate among 4-month-olds coconut did not differ statistically from that of 3 (age3 - age4: *P* = 0.7248) and 2-month-olds (age2 - age4: *P* = 0.9994). A statistically significant difference was found only when comparing the abortions of fruit of 3 and 5 months (age3 - age5: *P* = 0.0165) (Fig. 6B).

**Fig. 7:**
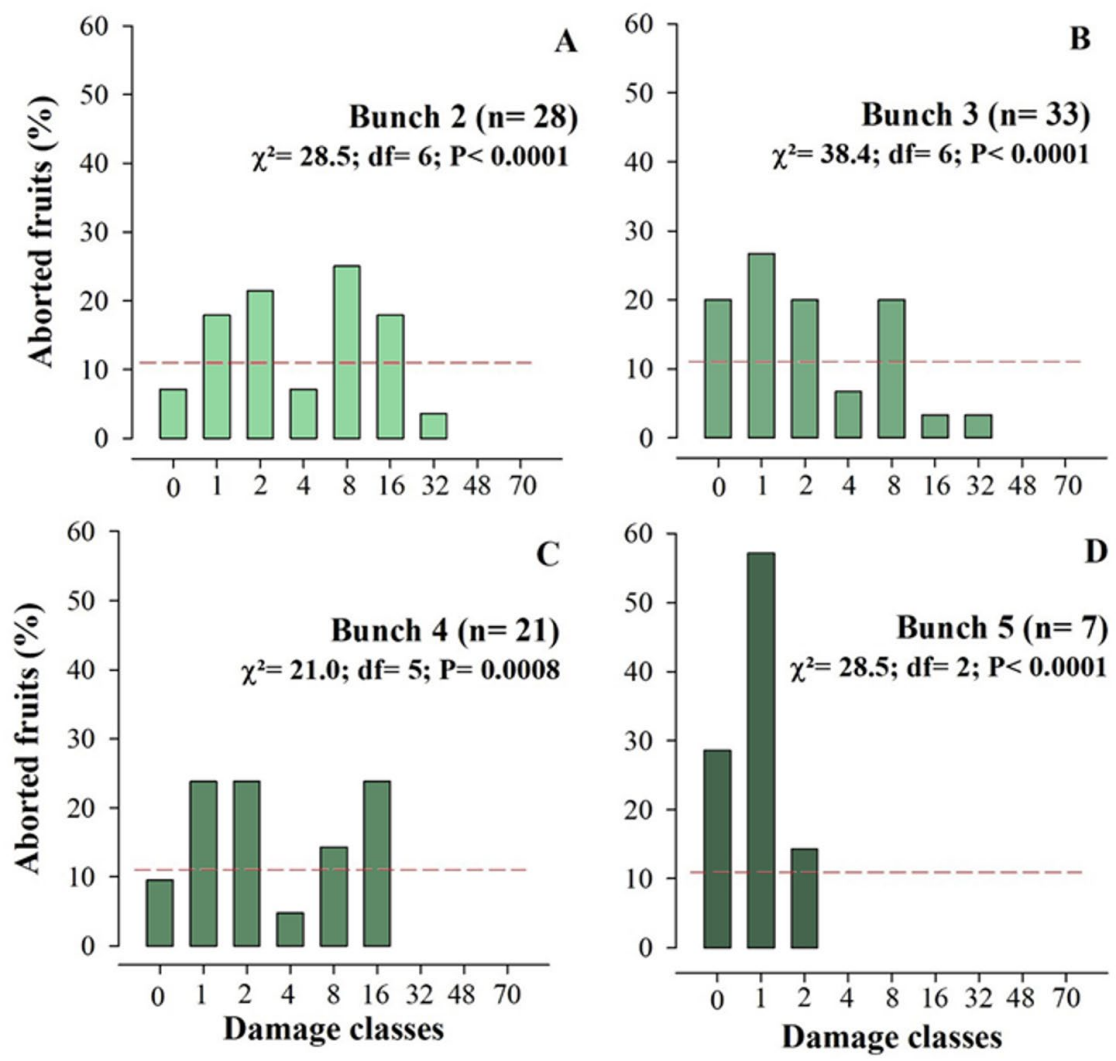
Damage intensity (chlorosis and/or necrosis) in aborted *Cocos nucifera* fruits artificially infested at different ages. The dashed red line represents the expected frequency of fruit abortion. **A**: 2 months; **B**: 3 months; **C**: 4 months; **D**: 5 months

The patterns in the frequency of damage caused by *A. guerreronis* in aborted fruits of different ages showed a trend towards fruit drop with low damage level of this mite (Figs. 7A–D). In general, fruit with mild necrosis (ratings 1 and 2) exhibited higher abortion rates, while more severe lesions were not associated with proportionally higher abortion rates. Additionally, fruit without visible lesions (rating 0) also frequently aborted, indicating natural fruit abortion. Despite this, for all ages, the observed frequency of different levels of damage to aborted fruits did not differ from the expected frequency, indicating randomness in the relationship between the damage level caused by *A. guerreronis* and coconut abortion (Figs. 7A–D).

## Discussion

Artificial infestation of coconut fruit with *A. guerreronis* revealed that: (1) the damage severity caused by *A. guerreronis* followed a sigmoidal growth pattern, increasing rapidly in younger fruit and slowing in older ones; (2) the cumulative number of aborted fruit increased almost linearly over the evaluation periods, reaching approximately 70% abortion by fruit maturity; (3) damage severity and abortion rates differed among the fruit age groups assessed; and (4) abortion rates were not directly associated with the severity of mite-induced damage.

Regression analysis demonstrated that *A. guerreronis* damage progresses rapidly in younger fruit and decelerates as fruit age increases. Consequently, the earlier the infestation occurs, the greater the potential impact on coconut production. The severity of damage caused by *A. guerreronis* may be related to the size of the mite population (Galvão et al. 2008; Sousa et al. 2017). Lima et al. (2012) showed that *A. guerreronis* typically initiates infestation in 2-month-old fruit, as access to the meristematic zone is constrained in younger fruit by the limited space between the floral bracts and the epicarp. The mite population peaks in fruit aged 3 to 4 months, averaging approximately 943 mites per fruit, followed by a gradual decline in older coconut fruit (Galvão et al. 2011).

Due to their limited mobility (Galvão et al. 2012; Melo et al. 2014), A. *guerreronis* disperses mainly through stochastic wind-driven events, which reduces the probability of individuals successfully locating and colonizing uninfested fruit. Therefore, for dispersal to be efficient, it is expected that a single female mite must be capable of founding a new colony (Navia et al. 2013; Galvão et al. 2012). Within approximately two months, *A. guerreronis* establishes itself and reaches its population peak in young fruit, demonstrating rapid development under favorable conditions.

Our results showed that when *A. guerreronis* infestation begins in older coconut fruits, necrosis growth occurs more slowly. This may indicate that older fruits are less suitable for the development of this mite. Older coconut fruit (> 5 months of age) present wider spacing between the bracts and epicarp, theoretically facilitating access to the meristematic zone by *A. guerreronis*. However, we rarely observed early mite damage in older fruit, which may corroborate the idea that these fruits are less suitable for establishment and population growth of *A. guerreronis*. The decline in *A. guerreronis* populations in older coconut fruit may result from both bottom-up and top-down regulatory mechanisms. In the former, reduced nutritional quality or higher concentrations of defense-related metabolites may slow the pest’s development in older fruit. Galvão et al. (2011) reported higher lignin content in older coconut fruit, the authors did not demonstrate how this might affect the development of *A. guerreronis*. To date, there are no studies evaluating the impact of coconut fruit chemical composition on the fitness of *A. guerreronis*, and thus this question remains open for further investigation.

In the latter case, population decline may be due to both direct and indirect action of predators. As coconut fruit mature, the bract-epicarp gap widens, facilitating predator access to the meristematic zone (Lima et al. 2012). Silva et al. (2016) demonstrated that *A. guerreronis* populations can be reduced by manipulating this access opening to allow the entry of the predatory mite *Neoseiulus paspalivorus* De Leon. Additionally, *A. guerreronis* can detect chemical cues from predators such as *Neoseiulus baraki* (Athias-Henriot) and *Amblyseius largoensis* (Muma) (Calvet et al. 2018). As such, the presence of predators in older fruit may trigger the dispersal of *A. guerreronis* individuals to younger fruit, where predators are less likely to reach the meristematic zone. Hence, the slower increase in necrotic area observed in older fruit may be a consequence of lower *A. guerreronis* densities, influenced by the aforementioned factors. This could help explain why the mite does not preferentially select older fruit (e.g., 5 months old) to initiate a colony. However, more detailed studies are needed to clarify this issue.

The observed frequency of coconut fruit abortion rates at different ages (young and mature) did not differ from the expected frequency regardless of the intensity of damage caused by *A. guerreronis*. This result indicates that the level of damage caused by this mite does not influence coconut fruit drop. Some studies report that infestation by *A. guerreronis* increases coconut fruit drop. (Moore et al. 1989; Seguni 2002; Rethinam et al. 2003; Rezende et al. 2016). All these studies only link the presence of mite damage in fallen fruit to the cause of abortion; however, multiple factors can cause premature fruit drop. Fruit abortion is a complex phenomenon that may also involve other factors, such as the interaction of *A. guerreronis* with other pests, as discussed by Paz-Neto et al. (2020; 2022). In addition to arthropod–plant interactions that may exacerbate fruit abortion, it is important to consider that abiotic factors may also contribute. Coconut palms require optimal conditions including temperatures of 27–29 °C, relative humidity below 80%, and annual rainfall around 1,500 mm. Deviations from these edaphoclimatic conditions can impact plant physiology and consequently induce fruit abortion (Fontes and Ferreira 2006; Lacerda et al. 2024).

Moreover, we observed that younger fruit (2, 3, and 4 months old) aborted more frequently than 5-month-old fruit. Abortion of vegetative or reproductive structures in response to herbivory is a known plant defense mechanism, allowing for optimized resource allocation (Patharkar et al. 2017; Strauss and Zangerl 2002). In this context, the abortion of infested young fruit may represent a physiological response by the coconut palm to mite attack. Redirecting resources to older infested fruit may offer an adaptive advantage to *C. nucifera*, as fruit damaged by *A. guerreronis* that reach maturity often show similar vigor to those not infested (Thomas et al. 2004; Beevi et al. 2006).

At this point, it is also important to note that, as part of the experimental protocol, only the selected fruit were retained in each bunch, and all other spikelets were removed at the beginning of the experiment. Although this procedure was intended to standardize resource allocation among the monitored fruit, such pruning may slightly modify within-bunch conditions, such as local source–sink balance or microenvironmental structure, which could influence fruit development and abortion. The potential magnitude of these effects may be better investigated in future studies. Therefore, the results presented here should be interpreted with caution.

Controlling *A. guerreronis* remains a major challenge for coconut producers. Because the mite develops in a protected area, pesticide sprays often have low efficacy (Silva et al. 2017). Moreover, as mentioned above, the narrow access to the meristematic zone in early fruit development stages limits the effectiveness of biological control agents (Lawson-Balagbo et al. 2008; Lima et al. 2012; Silva et al. 2016). Considering that this mite establishes colonies in fruit aged 2–3 months, and that early infestation leads to greater production losses, strategies that block *A. guerreronis* access to the fruit perianth may offer a promising avenue for pest management. Increasing the frequency of acaricide applications may help reduce mite dispersal, but this also raises production costs.

Our findings show that timing is a critical factor in minimizing losses caused by coconut mite infestation. Younger fruit subjected to infestation exhibited higher damage severity and abortion rates compared to older fruit. However, mite control alone may not prevent fruit abortion, as this process can also occur naturally due to various biotic and abiotic factors.

## Data Availability

The raw data that support the findings of this study have been deposited in Zenodo and are publicly available at: https://doi.org/10.5281/zenodo.18179112.
